# Supplementary material for: The 26S Proteasome Switches between ATP-Dependent and -Independent Mechanisms in Response to Substrate Ubiquitination
Source: Biomolecules. 2022 May 26;12(6):750. doi: 10.3390/biom12060750 (PMC9220805; doi:10.3390/biom12060750)
Supplement: Supplementary file 1 [file biomolecules-12-00750-s001.zip › biomolecules-1722247-supplementary.pdf]

The 26S Proteasome Switches Between ATP-Dependent and -Independent Mechanisms in Response to Substrate Ubiquitination

Abramo J. Manfredonia, Daniel A. Kraut

Supplementary Materials

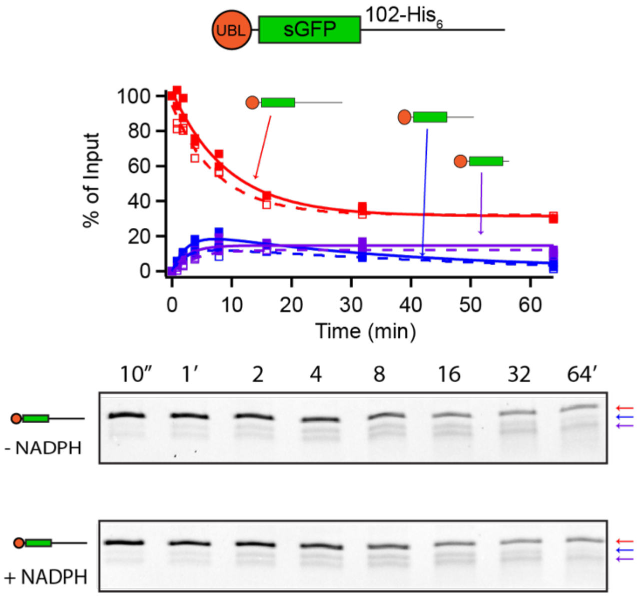

Supplementary Figure S1. The proteasome is unaffected by NADPH. Degradation of 20 nM UBL-sGFP-102-His$_6$ minus (solid symbols, solid fits) or plus (open symbols, dashed fits) 500 µM NADPH. Red is full-length substrate, blue is a partially truncated substrate that is initially formed and then degraded, and purple is a further truncated substrate that persists. Fits are to single or double exponentials. Protein is visualized and quantified using in-gel fluorescence of GFP.

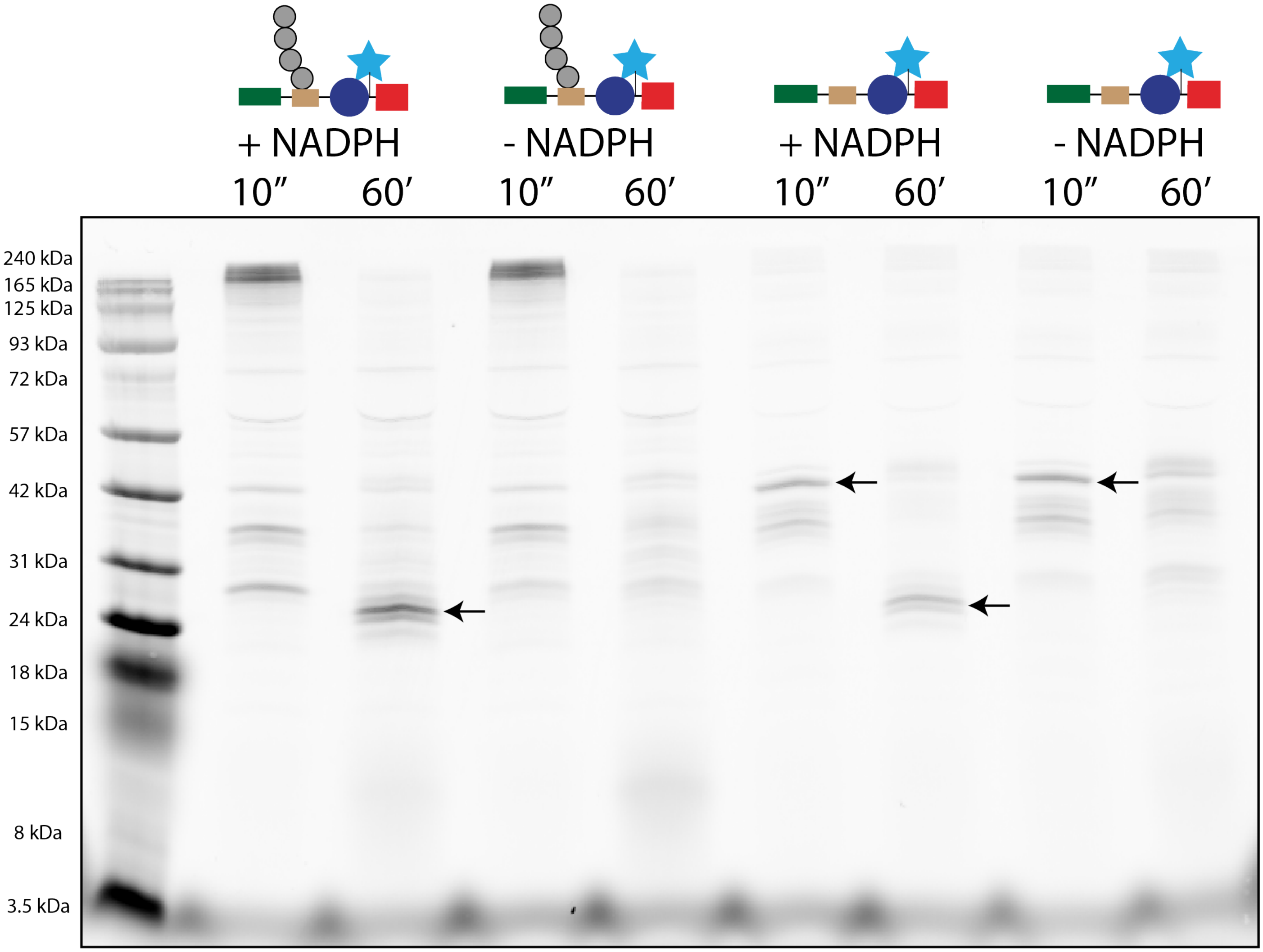

Supplementary Figure S2. Sizes of substrates and products of proteasomal degradation reactions compared with BlueStain 2 protein ladder (GoldBio) on a tris-tricine gel. Approximate MWs of ladder are given. Degradation of 20 nM Rpn4$^{1\text{-}80}$-PPXY-BarnaseΔKL89G-C-DHFRkΔC by 100 nM proteasome. Arrows indicate the size of full-length (non-ubiquitinated) substrate (predicted MW 40.8 kDa) and DHFR-containing fragment (predicted MW for DHFR plus a residual tail of 40-80 amino acids is 23-27 kDa.

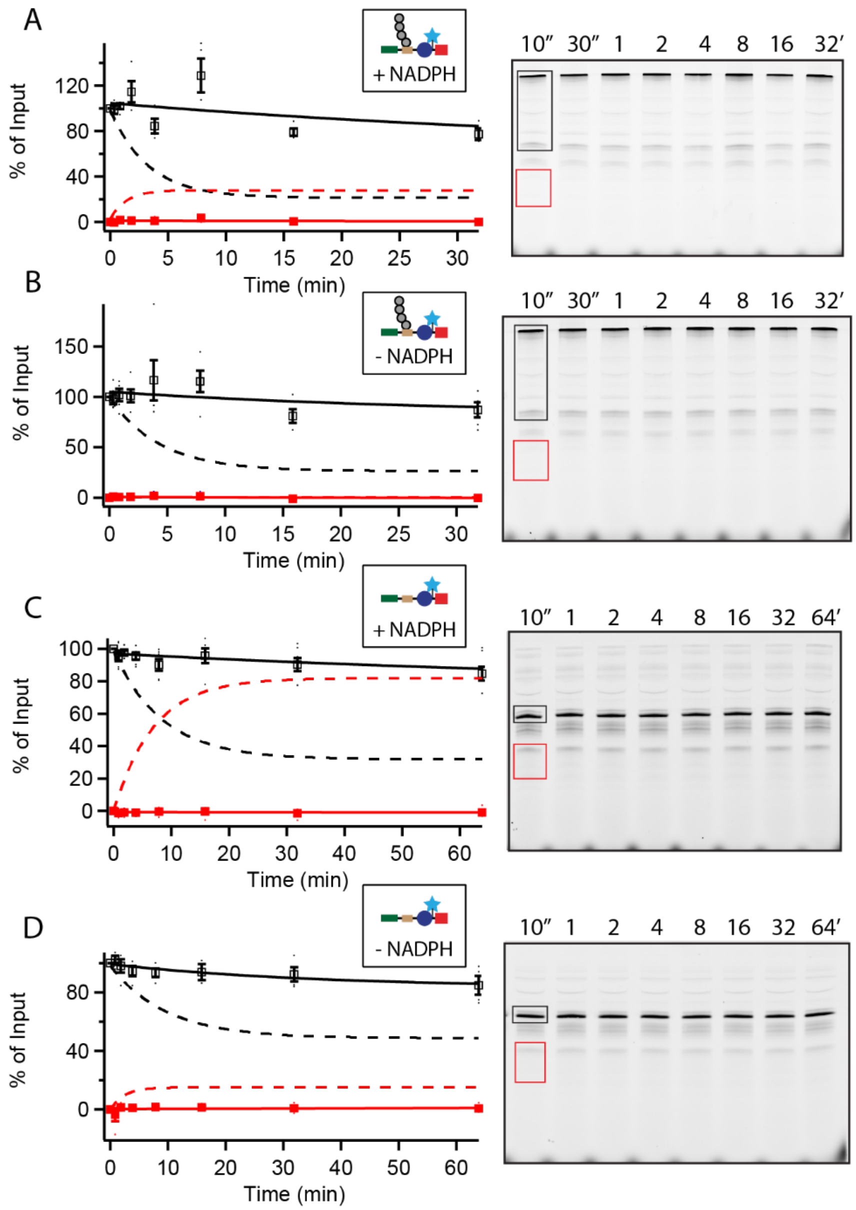

Supplementary Figure S3. Degradation of Rpn4-containing substrates is proteasome-dependent. (A-D) Degradation of 20 nM Rpn4$^{1\text{-}80}$-PPXY-BarnaseΔKL89G-C-DHFRkΔC in the absence of proteasome. Example gels show full-length substrate, (A-B) ubiquitinated or (C-D) non-ubiquitinated, outlined in black, and DHFR fragment outlined in red. Full-length (open squares) and DHFR fragment (closed squares) are shown as a percentage of total full-length present at the beginning of the reaction; full length is quantified as the sum of ubiquitinated and non-ubiquitinated substrate so any deubiquitination isn't misinterpreted as degradation. Dots are results from individual experiments and error bars represent the SEM of 4-6 experiments. Dashed lines are fits in the presence of WT proteasome from Figure 1.

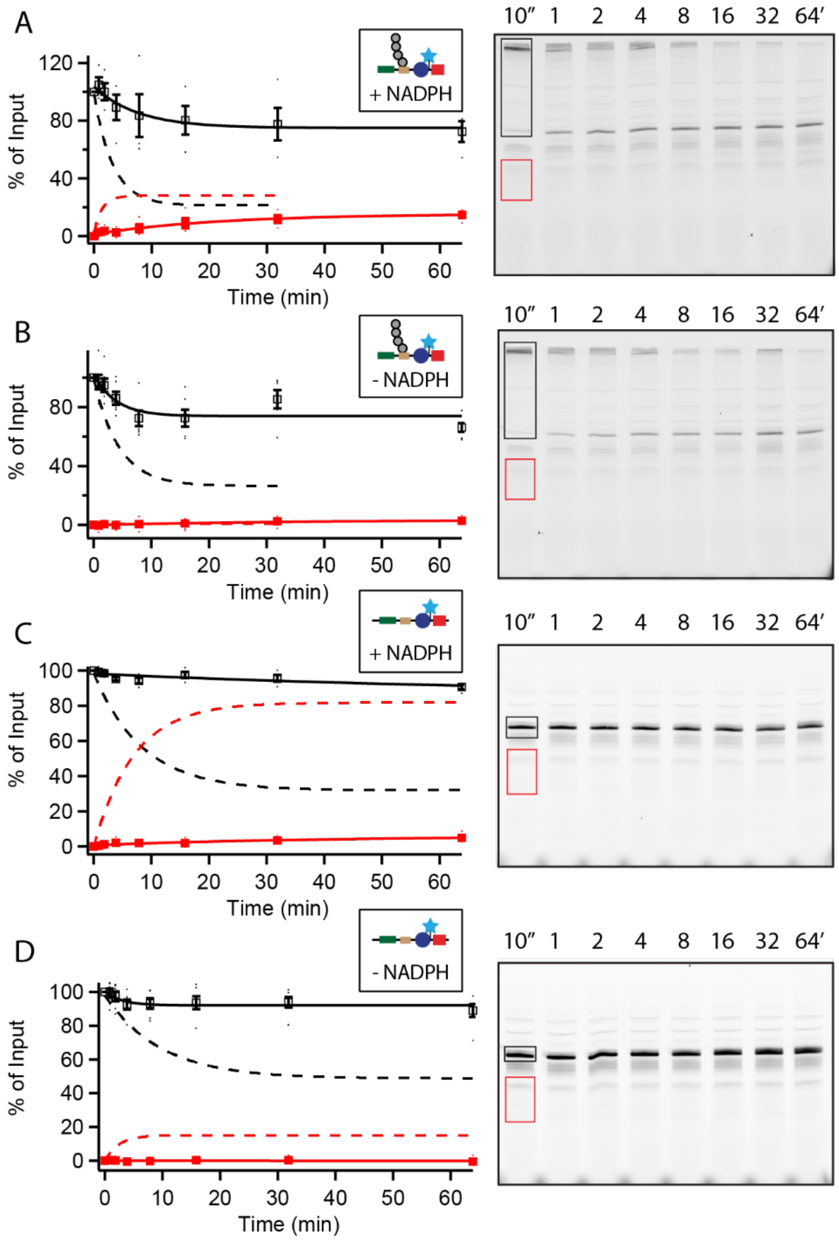

Supplementary Figure S4. Degradation of Rpn4-containing substrates is prevented by proteasome inhibitors. (A-B) Degradation of 20 nM Rpn4$^{1-80}$-PPXY-BarnaseΔKL89G-C-DHFRkΔC by 100 nM WT proteasome in the presence of proteasome inhibitors (100 µM each bortezomib, MG-132 and epoxomicin). Example gels show full-length substrate, (A ubiquitinated or (B) non-ubiquitinated, outlined in black, and DHFR fragment outlined in red. Full-length (open squares) and DHFR fragment (closed squares) are shown as a percentage of total full-length present at the beginning of the reaction; full length is quantified as the sum of ubiquitinated and non-ubiquitinated substrate so any deubiquitination isn't misinterpreted as degradation. Dots are results from individual experiments and error bars represent the SEM of 4-6 experiments. Dashed lines are fits in the presence of WT proteasome from Figure 1.

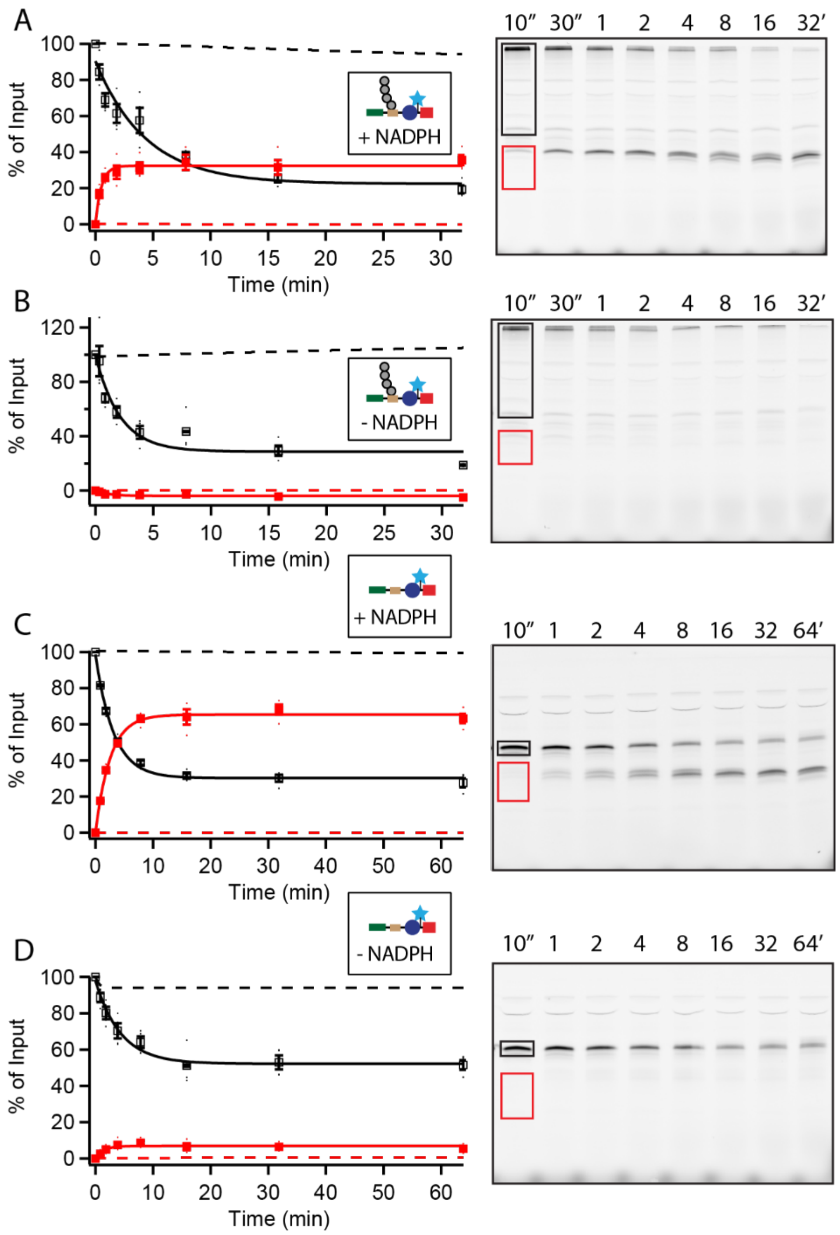

Supplementary Figure S5. Ubiquitin-independent degradation is universally less robust. (A-D) Degradation of 20 nM ubiquitinated (A, B) or non-ubiquitinated (C, D) yODC$^{1-44}$-PPXY-BarnaseΔKL89G-C-DHFRkΔC by 100 nM WT proteasome in the presence (A, C) or absence (B, D) of 500 µM NADPH. Example gels show full-length substrate outlined in black, and DHFR fragment outlined in red. Full-length (open squares) and DHFR fragment (closed squares) are shown as a percentage of total full-length present at the beginning of the reaction; full length is quantified as the sum of ubiquitinated and non-ubiquitinated substrate so any deubiquitination isn't misinterpreted as degradation. Dots are results from individual experiments and error bars represent the SEM of 4 experiments. Dashed lines are fits in the absence of proteasome from Figure S6.

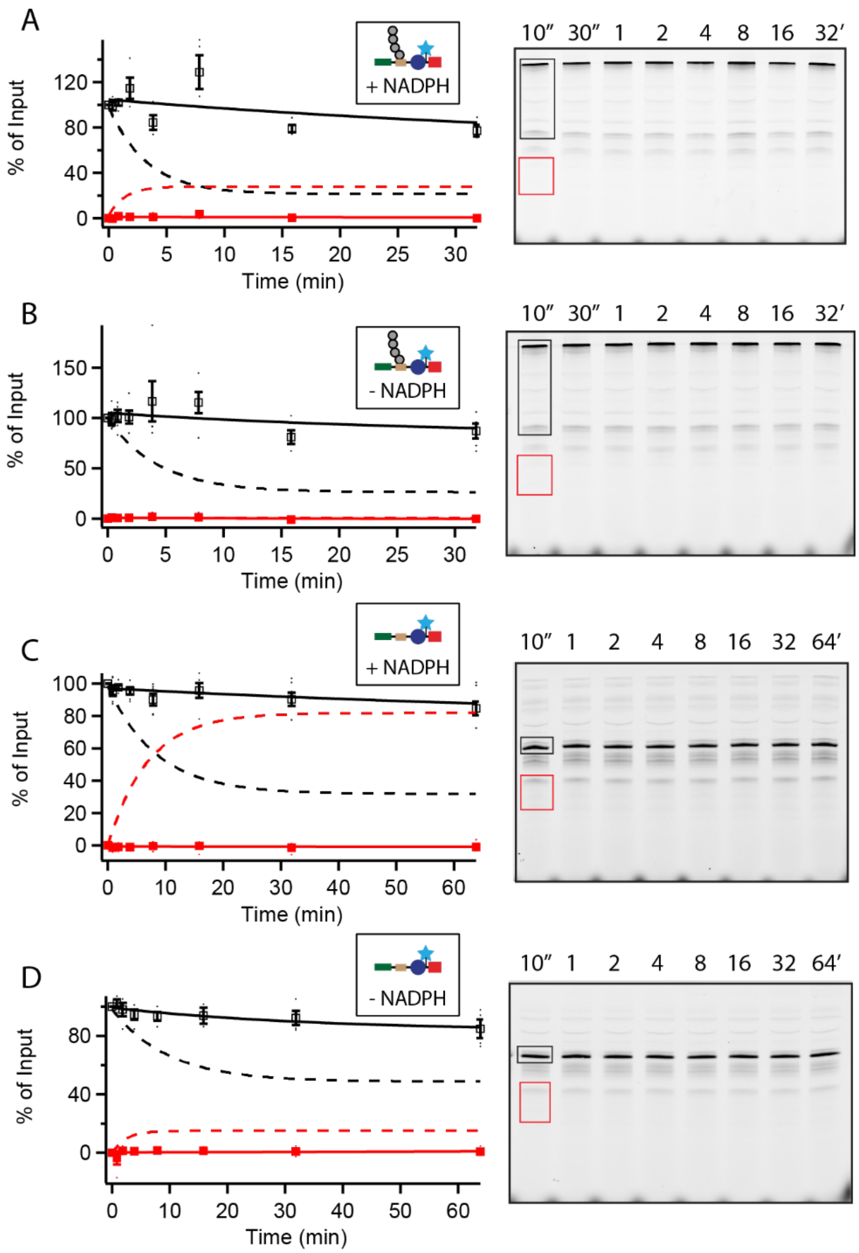

Supplementary Figure S6. Degradation of yODC-containing substrate is proteasome-dependent. (A-D) Degradation of 20 nM yODC$^{1\text{-}44}$-PPXY-BarnaseΔKL89G-C-DHFRkΔC in the absence of proteasome. Example gels show full-length substrate, (A-B) ubiquitinated or (C-D) non-ubiquitinated, outlined in black, and DHFR fragment outlined in red. Full-length (open squares) and DHFR fragment (closed squares) are shown as a percentage of total full-length present at the beginning of the reaction; full length is quantified as the sum of ubiquitinated and non-ubiquitinated substrate so any deubiquitination isn't misinterpreted as degradation. Dots are results from individual experiments and error bars represent the SEM of 3-4 experiments. Dashed lines are fits in the presence of WT proteasome from Supplementary Figure S5.

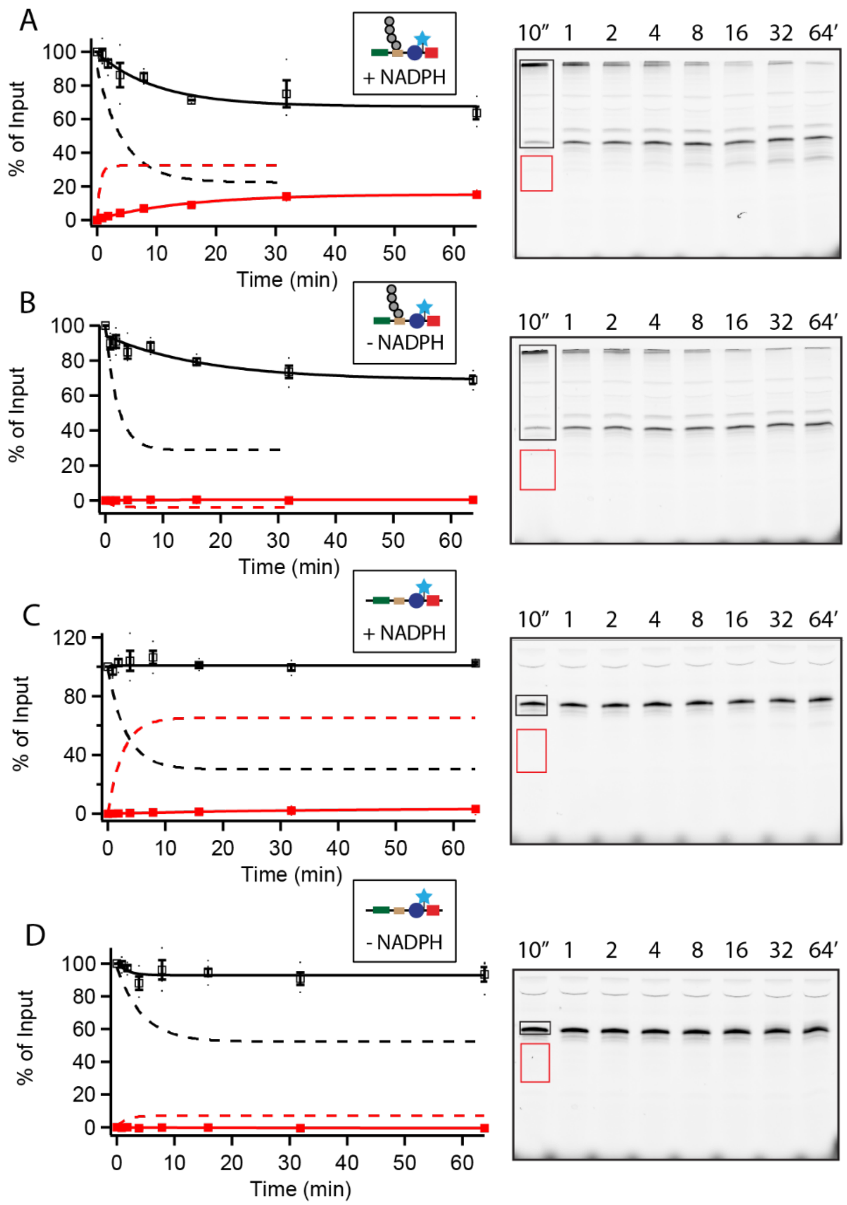

Supplementary Figure S7. Degradation of yODC-containing substrate is prevented by proteasome inhibitors. (A-B) Degradation of 20 nM yODC$^{1\text{-}44}$-PPXY-BarnaseΔKL89G-C-DHFRkΔC by 100 nM WT proteasome in the presence of proteasome inhibitors (100 µM each bortezomib, MG-132 and epoxomicin). Example gels show full-length substrate, (A ubiquitinated or (B) non-ubiquitinated, outlined in black, and DHFR fragment outlined in red. Full-length (open squares) and DHFR fragment (closed squares) are shown as a percentage of total full-length present at the beginning of the reaction; full length is quantified as the sum of ubiquitinated and non-ubiquitinated substrate so any deubiquitination isn' misinterpreted as degradation. Dots are results from individual experiments and error bars represent the SEM of 4 experiments. Dashed lines are fits in the presence of WT proteasome from Supplementary Figure S5.

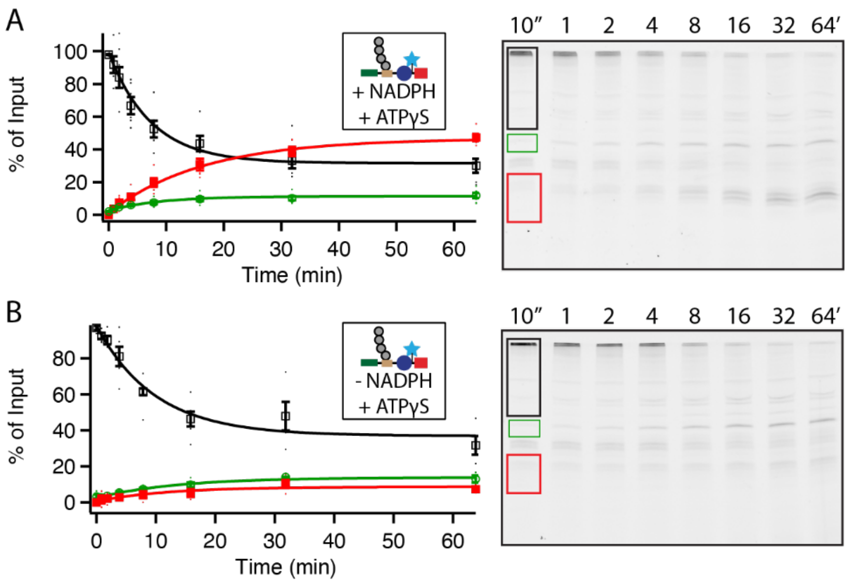

Supplementary Figure S8. Deubiquitination of ubiquitinated substrate in the presence of ATPγS. Data from Figure 2A, B were reanalyzed to examine the extent of deubiquitination of 20 nM Rpn4$^{1-80}$-PPXY-BarnaseΔKL89G-C-DHFRkΔC by 100 nM WT proteasome in the presence of ATP-γS in the presence (A) or absence (B) of 500 µM NADPH. The extent of deubiquitination was ~10% in both cases, with ~50-60% of the substrate being partially or fully degraded. Fits are to single exponentials.

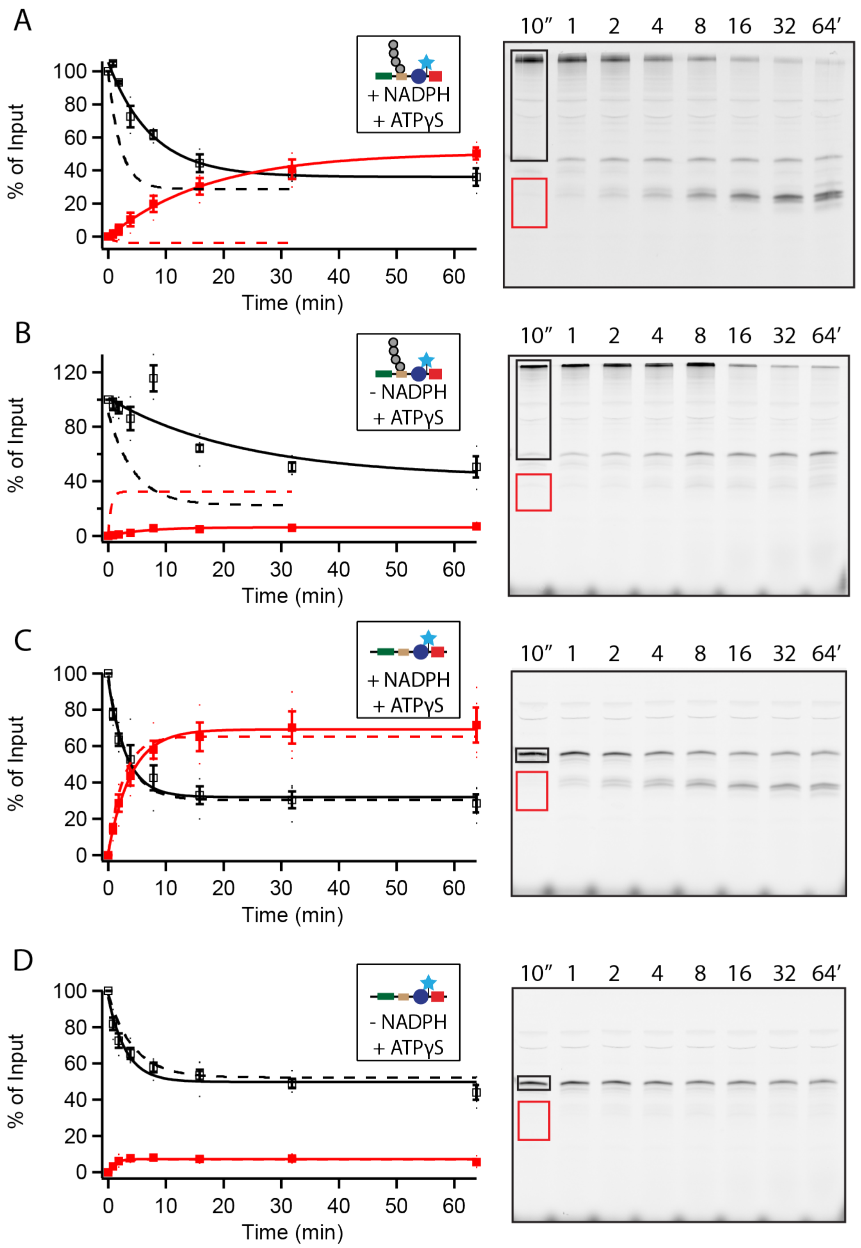

Supplementary Figure S9. Degradation of yODC-containing substrates is ATP-independent. (A-D) Degradation of 20 nM yODC$^{1-44}$-PPXY-BarnaseΔKL89G-C-DHFRkΔC by 100 nM WT proteasome in the presence of ATP-γS. Example gels show full-length substrate, (A-B) ubiquitinated or (C-D) non-ubiquitinated, outlined in black, and DHFR fragment outlined in red. Full-length (open squares) and DHFR fragment (closed squares) are shown as a percentage of total full-length present at the beginning of the reaction; full length is quantified as the sum of ubiquitinated and non-ubiquitinated substrate so any deubiquitination isn't misinterpreted as degradation. Dots are results from individual experiments and error bars represent the SEM of 3-4 experiments. Dashed lines are fits in the absence of ATP-γS from Supplementary Figure S5

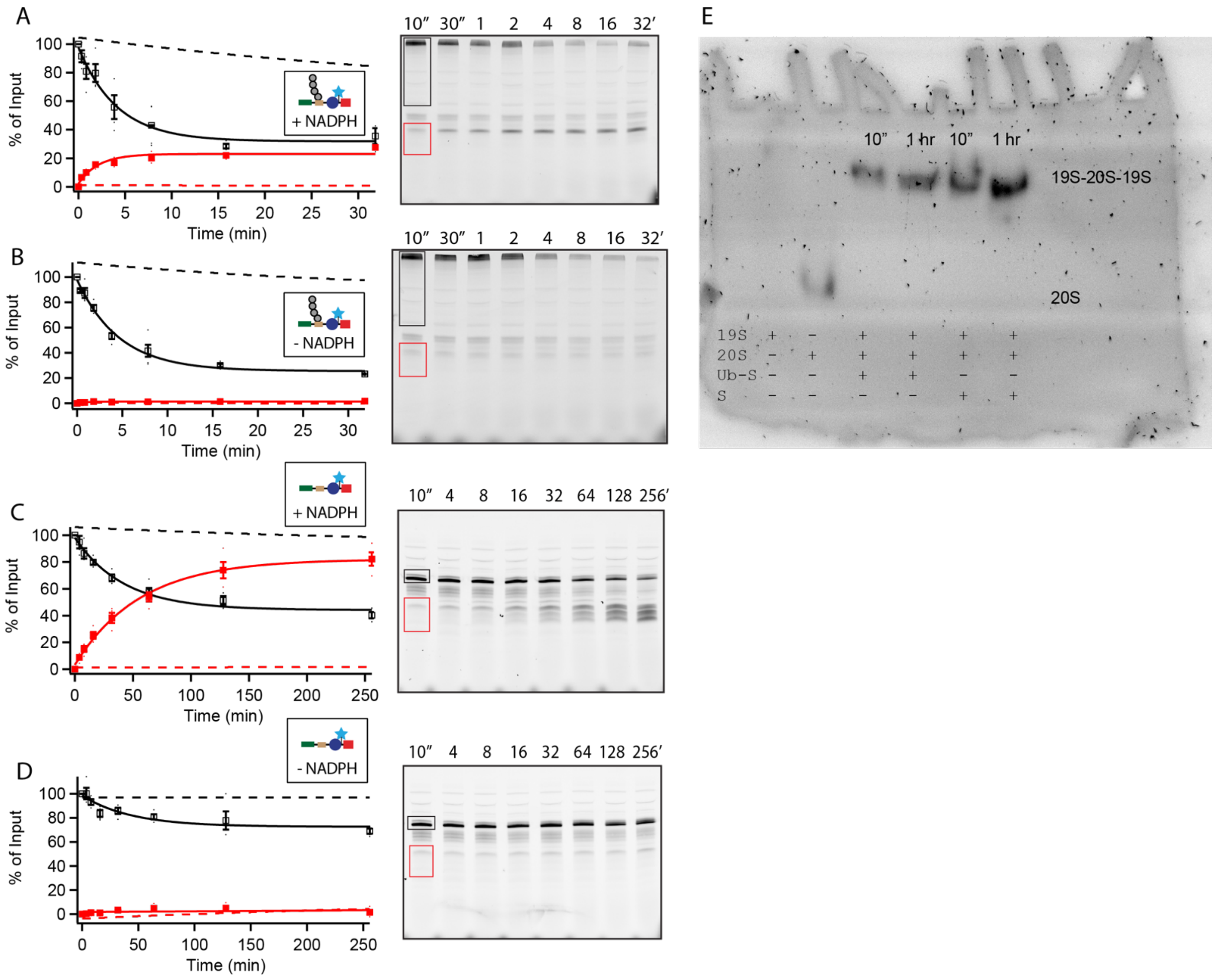

Supplementary Figure S10. Degradation of Rpn4-containing substrates is due to 26S proteasome. (A-D) Degradation of 20 nM Rpn4$^{1-80}$-PPXY-BarnaseΔKL89G-C-DHFRkΔC by 25 nM 20S + 75 nM 19S proteasome. Example gels show full-length substrate, (A-B) ubiquitinated or (C-D) non-ubiquitinated, outlined in black, and DHFR fragment outlined in red. Full-length (open squares) and DHFR fragment (closed squares) are shown as a percentage of total full-length present at the beginning of the reaction; full length is quantified as the sum of ubiquitinated and non-ubiquitinated substrate so any deubiquitination isn't misinterpreted as degradation. Dots are results from individual experiments and error bars represent the SEM of 4 experiments. Dashed lines are fits in the absence of proteasome from either Figure S1 (A, B) or replicate experiments performed under the same conditions (C, D). E) Native gel (visualized using Suc-LLVY-AMC) of proteasomal reconstitution as in degradation assays. No 20S is apparent after reconstitution even after 1 hour of incubation at 30 ˚C with ubiquitinated (Ub-S) or non-ubiquitinated (S) Rpn4$^{1-80}$-PPXY-BarnaseΔKL89G-C-DHFRkΔC.

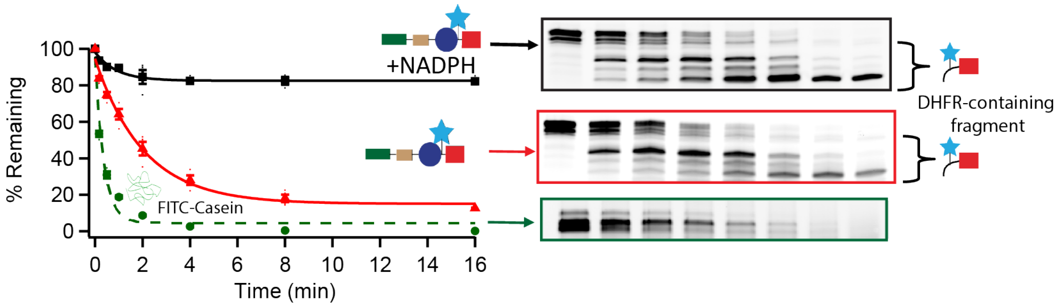

Supplementary Figure S11. Substrate is folded in native state and stabilized by NADPH. Proteolysis of 80 nM yODC$^{1\text{-}44}$-PPXY-BarnaseΔKL89G-C-DHFRkΔC (black squares, red triangles) and 2.5 µg/mL FITC-Casein (green circles) by 0.0125 mg/mL Thermolysin at 30°C. Example gels show full-length, non-ubiquitinated substrate in the presence (outlined in black) and absence (outlined in red) of NADPH, and FITC-casein (outlined in green). Full length substrate is shown as a percentage of total full-length present before the addition of Thermolysin. Dots are results from individual experiments and error bars represent the SEM of 4 experiments. FITC-casein data is taken from Figure 3.

Supplementary Table S1. Sequences and MWs of proteins

Protein	Sequence	MW	Notes
Rpn4[1-80]-PPXY-BarnaseΔKL89G-C-DHFRkΔC	MASTELSLKRTLTDILEDELYHTNPGHSQFTSHYQNYHPNASITPYKLVNKNKENNTFTWNHSLQHQNESSAASIPPQQTPPPPYGSMVINTFDGVADYLQTYHRLPDNYITASEAQALGWVASAGNLADVAPGMSIGGDIFSNREGRLPGRSGRTWREADINYTSGFRNSDRIGYSSDWLIYMTTDAYQTFTRIRTGCHLEMISLIAALAVDRVIGMENAMPWNLPADLAWFRRNTLNKPVIMGRHTWESIGRPLPGRRNIILSSQPGTDDRVTWVKSVDEAIAAAGDVPEIMVIGGGRVYEQFLPRAQRLYLTHIDAEVEGDTHFPDYEPDDWESVFSEFHDADAQNSHSYSFEILERR	40.8 kDa	His-SUMO tag in original protein is removed during purification
yODC[1-44]-PPXY-BarnaseΔKL89G-C-DHFRkΔC	MSSTQVGNALSSSTTTLVDLSNSTVTQKKQYYKDGETLHNLLLEPPPPYGSMVINTFDGVADYLQTYHRLPDNYITASEAQALGWVASAGNLADVAPGMSIGGDIFSNREGRLPGRSGRTWREADINYTSGFRNSDRIGYSSDWLIYMTTDAYQTFTRIRTGCHLEMISLIAALAVDRVIGMENAMPWNLPADLAWFRRNTLNKPVIMGRHTWESIGRPLPGRRNIILSSQPGTDDRVTWVKSVDEAIAAAGDVPEIMVIGGGRVYEQFLPRAQRLYLTHIDAEVEGDTHFPDYEPDDWESVFSEFHDADAQNSHSYSFEILERR	36.4 kDa	His-SUMO tag in original protein is removed during purification
His-SUMO	MGHHHHHHGSLQDSEVNQEAKPEVKPEVKPETHINLKVSDGSSEIFFKIKKTTPLRRLMEAFAKRQGKEMDSLRFLYDGIRIQADQAPEDLDMEDNDIIEAHREQIGG	12.4 kDa	Fused to N-termius of above proteins before purification
UBL-sGFP-102-His_6	MVSLTFKNFKKEKVPLDLEPSNTILETKTKLAQSISCEESQIKLIYSGKVLQDSKTVSECGLKDGDQVVFMVSQKKVDGGSGGGSMVSKGEELFTGVVPILVELDGDVNGHKFSVRGEGEGDATNGKLTLKFICTTGKLPVPWPTLVTTLTYGVQCFSRYPDHMKQHDFFKSAMPEGYVQERTITFKDDGTYKTRAEVKFEGDTLVNRIELKGIDFKEDGNILGHKLEYNFNSHNVYITADKQKNGIKANFKIRHNVEDGSVQLADHYQQNTPIGDGPVLLPDNHYLSTQSKLSKDPNEKRDHMVLLEFVTAAGITHGMDELYKLQLRYQPLLRISQNCEAAILRASQTRLNTIGAYGSTVPRSQSFEQDSRQRTQSWTALRVGAIPAATSSVAYLNWHNGQIDNEPQLDTNRQRILEGMGSMGMGHHHHHH	48.2 kDa	
